# Effect of Intranodally Administered Dendritic Cell-Based HIV Vaccine in Combination With Pegylated Interferon α-2a on Viral Control Following ART Discontinuation: A Phase 2A Randomized Clinical Trial

**DOI:** 10.3389/fimmu.2021.767370

**Published:** 2021-11-11

**Authors:** Lorna Leal, Elvira Couto, Sonsoles Sánchez-Palomino, Núria Climent, Irene Fernández, Laia Miralles, Yolanda Romero, Tania González, Maria José Maleno, Blanca Paño, Judit Pich, Carlos Nicolau, José Maria Gatell, Montserrat Plana, Felipe García, the DCV3-RISVAC04 Study Group, Lorna Leal

**Affiliations:** ^1^ Infectious Diseases Department—HIV Unit, Hospital Clínic Barcelona, Barcelona, Spain; ^2^ AIDS and HIV Infection Research Group, Institut d’Investigacions Biomèdiques August Pi i Sunyer (IDIBAPS), Barcelona, Spain; ^3^ Faculty of Medicine, Universitat de Barcelona, Barcelona, Spain; ^4^ Diagnostic Imaging Center (CDI), Hospital Clínic Barcelona, Barcelona, Spain; ^5^ Clinical Trials Unit (CTU), Hospital Clínic Barcelona, Barcelona, Spain; ^6^ ViiV Healthcare, Barcelona, Spain

**Keywords:** therapeutic vaccine, dendritic cell, interferon alpha, combined strategy, ATI

## Abstract

**Introduction:**

Functional cure has been proposed as an alternative to lifelong antiretroviral therapy and therapeutic vaccines represent one of the most promising approaches.

**Materials and Methods:**

We conducted a double-blind randomized placebo-controlled clinical trial to evaluate the safety, immunogenicity, and effect on viral dynamics of a therapeutic vaccine produced with monocyte-derived dendritic cells (MD-DC) loaded with a high dose of heat-inactivated autologous (HIA) HIV-1 in combination with pegylated interferon alpha 2a (IFNα-2a) in people with chronic HIV-1.

**Results:**

Twenty-nine male individuals on successful ART and with CD4+ ≥450 cells/mm^3^ were randomized 1:1:1:1 to receive three ultrasound-guided inguinal intranodal immunizations, one every 2 weeks: (1) vaccine ~10^7^ MD-DC pulsed with HIA-HIV-1 (10^10^ HIV RNA copies) (*n* = 8); (2) vaccine plus three doses of 180 mcg IFNα-2a at weeks 4–6 (*n* = 6); (3) placebo = saline (*n* = 7); and (4) placebo plus three doses of 180 mcg IFNα-2a (*n* = 8). Thereafter, treatment was interrupted (ATI). Vaccines, IFNα-2a, and the administration procedures were safe and well tolerated. All patients’ viral load rebounded during the 12-week ATI period. According to groups, changes in viral set-point between pre-ART and during ATI were not significant. When comparing all groups, there was a tendency in changes in viral set-point between the vaccine group vs. vaccine + IFNα-2a group (>0.5log_10_ *p* = 0.05). HIV-1-specific T-cell responses (IFN-ƴ Elispot) were higher at baseline in placebo than in the vaccine group (2,259 ± 535 vs. 900 ± 200 SFC/10^6^ PBMC, *p* = 0.028). A significant difference in the change of specific T-cell responses was only observed at week 4 between vaccine and placebo groups (694 ± 327 vs. 1,718 ± 282 SFC/10^6^ PBMC, *p* = 0.04). No effect on T-cell responses or changes in viral reservoir were observed after INFα-2a administration.

**Discussion:**

Results from this study show that intranodally administered DC therapeutic vaccine in combination with IFNα-2a was safe and well-tolerated but had a minimal impact on viral dynamics in HIV-1 chronic infected participants.

**Clinical Trial Registration:**

(www.ClinicalTrials.gov), identifier NCT02767193

## Introduction

Despite the success of antiretroviral therapy (ART) in significantly reducing the morbidity and mortality of people living with HIV (PLWH) (1), this treatment has major limitations such as drug toxicities, high cost, and the need for lifetime adherence to prevent occurrence of resistance (2, 3). ART is not capable of restoring HIV immune-specific responses; in fact, it could have a negative impact in the specific T-lymphocyte response due to the lack of antigen exposure (4, 5). Another major limitation is that this treatment by itself is unable to eradicate the infection, mainly because ART cannot eliminate HIV reservoir. This reservoir consists of latently infected cells carrying replication-competent virus despite suppressed plasma viremia (6) and are responsible for viral rebound if ART is interrupted (7). It is necessary to search for effective alternatives to achieve a durable control of HIV replication.

Functional cure has been proposed as an alternative to “ART for life” and therapeutic vaccines represent one of the most promising approaches (8, 9). The goal of therapeutic vaccination is to augment virus-specific immune responses using a controlled exposure to HIV antigens. A therapeutic strategy that has been pursued is dendritic cell (DC)-based vaccines. DCs have unique characteristics that made them an excellent option for vaccines. They are considered the most effective antigen-presenting cells (APCs) responsible for primarily sensitizing naive T cells to specific antigens (10). There have been several differently designed clinical trials using DC-based therapeutic HIV vaccines with an overall good safety profile, able to boost immune responses against HIV-1 and some of them even had a significant effect on viral load set-point after ART interruption (11–13). These results encourages further research on this type of intervention and perhaps a different approach considering that a vaccine alone may not be sufficient to cure HIV-1 infection (14, 15).

One major obstacle to cure HIV is the latent reservoir. It has been reported that interferon alpha-based therapies reduce integrated HIV-DNA even after ART interruption (16). Also, it has been observed that interferon alpha (IFNα) can boost CD8^+^ T-cell responses (17). In cancer, combining IFNα with DC-based vaccines has shown to be safe and to boost immune-specific responses (18).

We report here the safety, immunogenicity, and effect on viral dynamics of an autologous DC-based vaccine administrated intranodally and combined with pegylated interferon alpha-2a (IFNα-2a) in people with chronic HIV-1 under standard ART.

## Materials and Methods

### Study Design and Interventions

**Figure 1:**
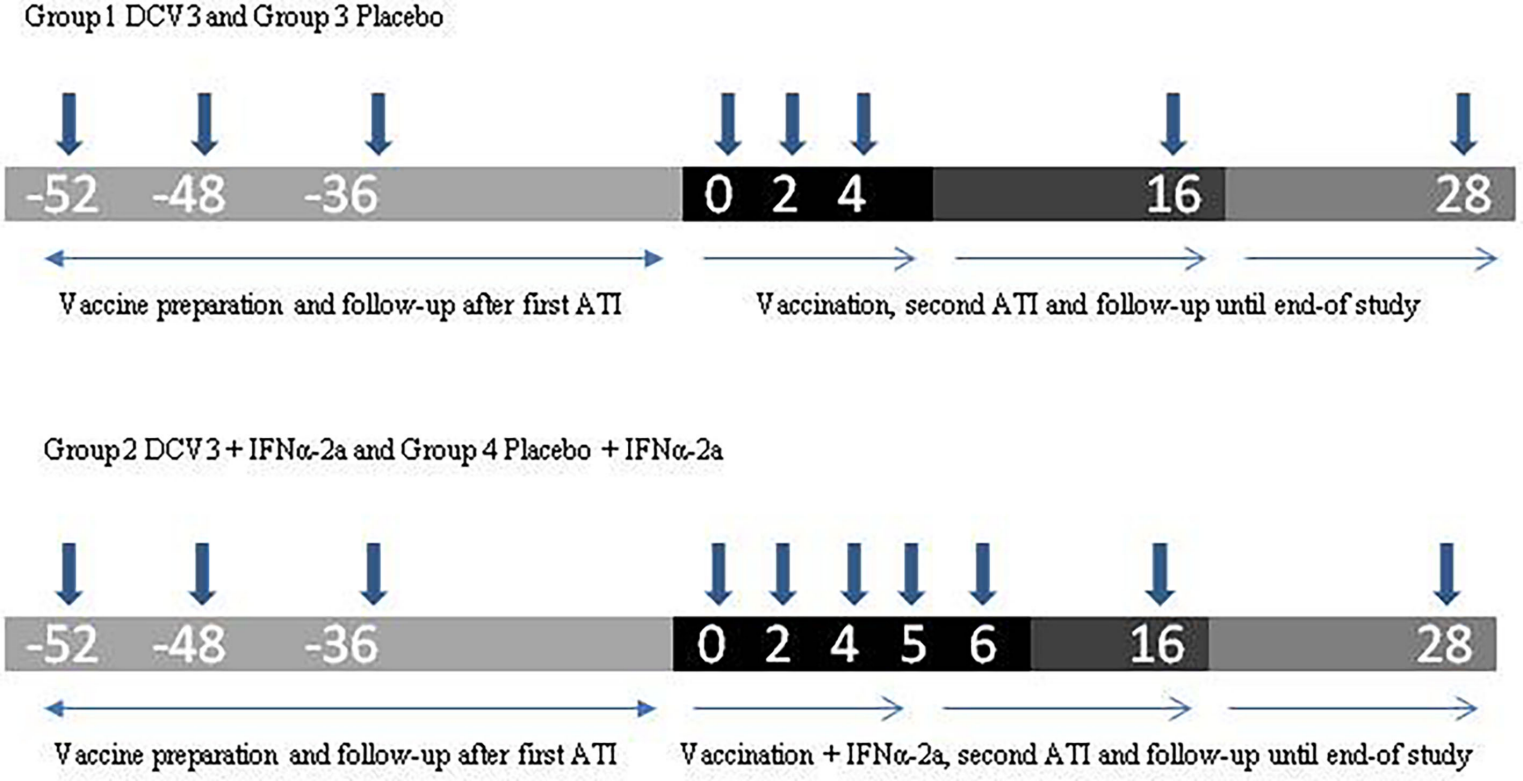
Study design.

This was a randomized, double-blind, placebo-controlled, single-center phase 1/2 clinical trial to assess the safety, immunogenicity, and virologic effect of a therapeutic vaccine prepared with autologous monocyte-derived DC (MC-DC) pulsed with a high titer of autologous heat-inactivated HIV-1 (DCV3) administered intranodally in combination with pegylated IFNα-2a. The study was divided into three phases (**Figure 1**):

Phase 1: Eligible participants were randomized to four study arms through the electronic clinical research file. Study arms were represented as follows: (1) DCV3 vaccine; (2) DCV3 vaccine + IFNα-2a; (3) Placebo; and (4) Placebo + IFNα-2a. The random list code was created by a computer program following a 1:1:1:1 pattern. This same program generated a unique code for every participant treatment. Vaccine/placebo treatment was double-blinded, but IFNα-2a administration was open-label. Individuals taking non-nucleoside reverse transcriptase inhibitors (NNRTIs) were switched to an integrase inhibitor-based regimen at least 2 weeks before treatment interruption.

Randomized participants discontinued ART (week −48) and were weekly followed-up until HIV-1 plasma viral load (pVL) were ≥5,000 copies/ml (approximately 4 weeks) and a 100 ml blood sample was obtained for the preparation of the inactivated autologous viral stock. Participants remained without ART until viral culture was confirmed and then restarted their previous treatment (week −36). Participants continued with phase 2 once they had at least two VL measures below the limits of assay detection during the following 9 months.

Phase 2: All participants received two ultrasound-guided intranodal injections (250 µl) containing DCV3 vaccine or placebo, each in a different inguinal node at weeks 0, 2, and 4. Participants allocated to study arms 2 and 4 also received 1 subcutaneous injection with 180 mcg pegylated IFNα-2a at weeks 4, 5, and 6.

Phase 3: Vaccinated participants with a fully suppressed pVL and a CD4 T-cell count ≥ 450 cells/mm^3^ interrupted ART at week 4. They were closely monitored weekly until week 6 and then bi-weekly during the next 10 weeks with measurements of CD4^+^ T cell counts and VL. Criteria to restart ART before ending the 16-week analytical treatment interruption (ATI) were as follows: a VL measure ≥ 100,000 copies/ml in two consecutive determinations, a confirmed >30% decline in CD4^+^ T cell count, or an absolute CD4^+^ T cell count <350 cells/mm^3^ or any other clinical condition that may suppose a risk according to investigators. Participants with a detectable VL at week 16 restarted ART. Participants with a fully suppressed VL at week 16 continued without ART and bi-weekly follow-up. Once participants restarted ART, they were followed up every 4 weeks during 12 weeks when the study ended.

### Study Subjects

Eligible participants were those with chronic HIV-1 under stable ART for at least 12 months, with baseline CD4^+^ T cells ≥ 450 cells/mm^3^, a CD4^+^ T cell nadir count ≥ 350 cells/mm^3^, and documented suppression of plasma viremia (at least 2 measures) below the limits of assay detection for at least 6 months before enrolment. Participants who had started ART with a suboptimal regimen (less than three active drugs), those who had any psychiatric clinical history, and those who had a low platelet count (<80,000/mm^3^) and hemoglobin below 12 g/dl were excluded. Participants were informed in detail about all procedures and study objectives and gave written informed consent. The clinical protocol was approved by the institutional ethical review board (CEIC) and by the Spanish Regulatory Authorities (AEMPS).

### Study Products

#### Preparation of Inactivated Autologous Viral Stocks for Pulsing Autologous MD-DC

For each participant exhibiting an HIV-1 VL ≥ 5,000 copies/ml after ART discontinuation, we drew 100 ml of peripheral blood EDTA-treated sample to obtain one lot of high-titer autologous inactivated HIV-1 stock that was prepared fulfilling clinical-grade good manufacturing practice (cGMP) requirements and complying with the predetermined specifications in the approved investigational medicine product dossier. Briefly, a primary virus isolate was obtained and propagated by equal ratio co-culturing enriched CD8^+^ T cells and macrophage derived from monocytes (CD14^+^ cells) in peripheral blood mononuclear cell (PBMC) samples from the participants. CD8^+^ T cells were 24 h *in vitro* activated in the presence of anti-CD3 and anti-CD28 and cultured with IL2. Mature macrophages (CD71^+^cells) were progressively detected in culture with a concomitant decline in CD14^+^. The virus-containing co-culture supernatants were collected at days 7 and 14 and heat-inactivated twice at 56°C for 30 min and concentrated by ultracentrifugation to a final volume of 1 ml, which was divided into five 0.2-ml aliquots and stored frozen at −80°C until use for pulsing immature MD-DC. The final products contained ≥10^7^ HIV-RNA copies/ml and a high infectivity reduction after heat treatment (mean value of 4.69 × 10^10^ ± 1.15 × 10^10^ copies/ml).

#### *In Vitro* Generation of MD-DC, Their Pulsing With Inactivated HIV-1 Under cGMP Conditions, and Immunizations

One week before administration of each dose of vaccine (weeks −1, 1, and 3), blood monocyte samples were obtained and cultured for 7 days to develop MD-DC. PBMCs were isolated, within 1 h of the blood extraction, from a 150 ml sample of EDTA-treated venous blood by means of standard Ficoll gradient centrifugation. After being washed four times in cGMP phosphate-buffered saline (PBS) (Lonza), PBMCs were resuspended in MD-DC culture medium including X-VIVO 15 culture medium (Lonza) supplemented with 1% heat-inactivated autologous serum and pharmaceutical gentamicin (B. Braun Medical Supplies), fungizone (Gibco-LifeTechnologies), and 1 mM zidovudine (GES Genéricos Españoles); settled in sterile apyrogenic culture flasks; and incubated for 2 h in a cell culture incubator (37°C, humidified atmosphere with 5% CO_2_ in air). Thereafter, nonadherent cells were discarded, and adherent cells (enriched monocytes) were immediately washed gently (three times) with cGMP PBS to eliminate residual contaminating lymphocytes, and then cultured for 5 days in MD-DC culture medium with recombinant human granulocyte macrophage colony-stimulating factor (GM-CSF) (1000 IU/ml) (CellGenix) and cGMP recombinant human interleukin-4 (IL-4) (1000 IU/ml) (CellGenix). Cytokines at the indicated doses were added at 2- to 3-day intervals. On day 5 of culture, immature MD-DCs were collected, washed (three times) with PBS, pulsed for 2 to 3 h with freshly thawed frozen aliquot of heat-inactivated autologous HIV-1 (0.2 ml containing >10^8^ HIV-1 copies/ml) in a final concentration of 2 × 10^7^ MD-DC/ml in MD-DC culture medium with GM-CSF and IL-4, and cultured in an ultralow attachment flasks to prevent cell adhesion. After viral pulsing, MD-DC were fully matured during a total of 48 h with a cocktail of cytokines containing IL-1β (300 IU/ml) (CellGenix), IL-6 (1,000 IU/ml) (CellGenix), tumor necrosis factor-α (1,000 IU/ml) (CellGenix), and prostaglandin E2 (1 mg/ml) (Dinoprostona, Pfizer) and MD-DC medium with GM-CSF and IL-4 was added to a concentration of 0.5 × 10^6^ MD-DC/ml. Immediately after pulsing with inactivated autologous HIV, cells were washed once and resuspended in 0.5 ml of pharmaceutical saline solution supplemented with 1% of pharmaceutical human albumin (Grifols, Barcelona, Spain) containing >10^6^ MD-DC and administered in less than 30 min (0.25 ml each injection). A small aliquot, removed immediately before injection, was used for immunophenotyping, Gram staining, and Mycoplasma detection. Before pulsing, microbiological culture was performed with uniform negative results. The amount of cells obtained and the maturation, as assessed by flow cytometry, was consistent throughout all the immunization periods.

#### Preparation of Placebo and Ultrasound-Guided Intranodal Administration

Placebo syringes were composed of 0.25 ml of sodium chloride at 1% of seric human albumin and were prepared by the hospital’s pharmacy.

All treated participants received two doses of vaccine/placebo each in a different inguinal lymph node at weeks 0, 2, and 4. Intranodal administration was ultrasound-guided and performed by a trained radiologist. Left and right inguinal areas were alternated.

#### Pegylated Interferon

Participants randomly allocated to study arms 2 and 4 received a 1 ml subcutaneous injection with 180 mcg of pegylated IFNα-2a (Pegasys^®^ Roche Registration Limited) at weeks 4, 5, and 6.

### Study Endpoints

There were two primary endpoints: safety and vaccine effect on HIV-1 viral load dynamics. Safety was assessed as the rate of occurrence of grade 3 or higher local and systemic AEs (including SAEs), which were at least possibly related to the procedures or study products. AEs were graded according to the Division of AIDS (DAIDS) Table for Grading the Severity of Adult and Pediatric Adverse Events, version 2.0, November 2014 (19). Main virologic outcome was the proportion of participants with a suppression of plasma viremia below the limits of assay detection after 12 weeks since discontinuation of ART.

Secondary endpoints were rate of occurrence of grade 1 and 2 local and systemic AEs, changes in the HIV-1-specific immune response measured by ELISPOT and changes in the viral reservoir between baseline and post-vaccination, and rate of participants with viral rebound during ATI. All the immunologic and virologic determinations were performed in a blinded fashion.

### Immunogenicity

Immunogenicity was assessed on cryopreserved PBMCs at screening, baseline (2 weeks before first immunization), and weeks 2, 4, 8, 16, and 28 of follow-up by the quantification of T-cell responses by an interferon-gamma (IFN-γ) ELISpot assay in a single research laboratory, as described before (13). ELISPOT assays were performed to measure the numbers of IFN-g-producing PBMCs directed against HIV-1 sequences. Briefly, assays were performed with cryopreserved PBMCs using 20 pools of peptides, consisting of 15-nucleotide oligomers overlapping by 11, grouped in pools of 10 to 12 peptides each, covering the whole HIV-1 subtype B proteome (five pools of Gag, six pools of Pol, one of Vif, five pools of Env, one of Nef, one of Vpu/Vpr, and one of Tat/Rev) from the NIH AIDS reagent program. Negative control responses were obtained with unstimulated cells. Positive controls included cells stimulated with phytohemagglutinin A (Sigma) and a CEF pool containing 32 HLA class I–restricted peptides from cytomegalovirus, Epstein–Barr virus, and flu virus (National Institutes of Health AIDS Research and Reference Research Program). All time points for an individual participant were tested in a single assay. The ELISPOT assay was done in 96-well polyvinylidene difluoride microtiter plates coated overnight with a monoclonal antibody (mAb) specific for human IFN-g (mAb 1-DK-1, Mabtech, Denmark). PBMCs resuspended in RPMI plus 10% fetal calf serum were plated in the presence of different peptide pools (2 µg/ml, final concentration) and incubated overnight at 37°C and 5% CO_2_. Plates were developed with biotinylated anti-human IFN-g, streptavidin conjugated to alkaline phosphatase (Mabtech), and chromogenic substrate bromochloroindolyl phosphate–nitro blue tetrazolium (Biorad). SFCs were counted with an AID ELISPOT reader (Autoimmun Diagnostika GmbH). Results were expressed as the number of SFCs per million of PBMCs after subtracting the background. The positivity threshold for each peptide pool or antigen was defined at 50 SFCs/106 PBMCs. At least 85% of the responding cells with this methodology are CD8^+^ T cells, but because all studies were conducted with unfractionated PBMCs, responses are described as HIV-specific T cells.

### Quantification of HIV DNA—Viral Reservoir

HIV reservoir was quantified as total and integrated HIV-1 DNA in isolated CD4^+^ T cells at screening, during first ATI before reintroduction of ART (w−36), 1 week before immunizations (w−1), and weeks 3, 16, and 28 of follow-up. Total CD4^+^ T cells were enriched from participants’ thawed PBMCs using negative selection magnetic beads (Stemcell Technologies, Vancouver, Canada). The pursued molecular form was amplified from CD4^+^ T-cell DNA lysates subjected to a first round of amplification [long terminal repeat (LTR)-gag amplification for total DNA and Alu-LTR amplification for integrated DNA], essentially as referred (20). These first amplicons were then subjected to a nested PCR with internal specific primers for each region using TaqMan probes. It is noteworthy to mention that HIV-1 sensitivity was three viral DNA copies. For each assay, the CD3^+^ gene copy number (two copies per cell) was determined in the same tube for accurate quantification of the total gene copy number. The HIV/CD3 copy ratio results in the frequency of cells harbouring HIV DNA. For total and integrated HIV DNA, the standard curve was elaborated with serial dilutions of ACH2 cells, carrying one single HIV provirus, ranging from 3 × 10^5^ to 3 cells together with the experimental samples. HIV primers and probes were optimized to efficiently amplify and detect HIV-1 from the A, B, C, D, and A/E (CRF01) clades.

### Statistical Analyses

All randomized participants were included in the safety analysis. Results are displayed as *n* (%), median, and interquartile range (IQR) unless stated otherwise. The primary safety endpoint is described by number and percentage of grade 3 or above AEs.

Sample size was calculated considering a type 1 error of 5% and a power of 80% taking into account that 50% of participants allocated to study arm 2 would have an undetectable pVL at week 16 and that no participant in the control arm would have an undetectable pVL at the same study week. A total number of eight participants in each arm were needed to detect such a difference.

The HIV RNA values were log^10^ transformed before analysis. Participants’ measurements were censored after ART initiation in those participants who had to start ART according to pre-established criteria. Baseline pVL and CD4^+^ T cell count were defined as the mean of all determinations available 1 year before any ART. The continuous variables were compared between groups with the nonparametric Mann–Whitney test. The continuous variables were compared within groups with the nonparametric Wilcoxon test. Categorical variables were compared between groups with the Fisher’s exact test. Changes in plasma HIV1 RNA viremia and the frequency of total HIV-specific T-cell responses over the 48 weeks of the trial were analyzed by an AUC measurement. Spearman rank order correlations were calculated to assess the correlation between continuous variables. Corrections were performed for multiple comparisons.

## Results

### Intranodal Vaccination With DCV3 in Combination With Pegylated IFNα-2a Is Safe and Well-Tolerated

**Figure 2:**
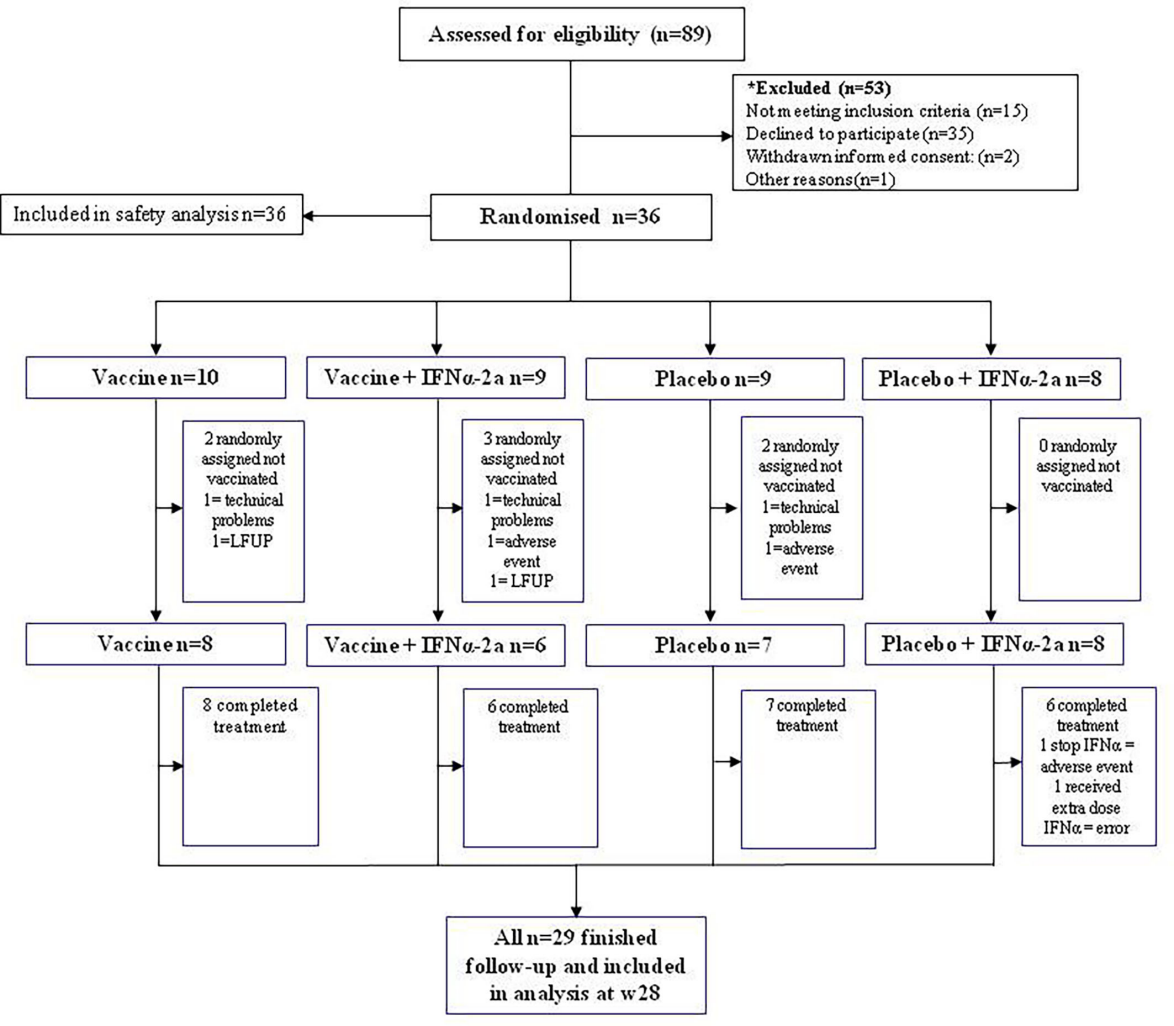
Consort diagram of participants’ distribution.

Between May 2016 and October 2017, a total of 36 PLWH living in Catalonia, Spain were enrolled and randomized. Seven participants that were randomized and went through first ATI did not receive any study products: three were excluded because culture virus isolation was not possible—two because the virus did not amplify and the other one because pVL did not rebound after 10 weeks since ART discontinuation; two were excluded because they presented severe unrelated adverse events—one was a participant with several comorbidities that had a subarachnoid hemorrhage and the other one was diagnosed with a seminoma; and two were lost to follow-up. A total of 29 participants completed the vaccination phase and were followed-up until the end of the study (**Figure 2**). Baseline characteristics and CD4 evolution of participants are shown on **Table 1**.

**Table 1:** Baseline characteristics.

Variables	All participants, *n* = 29 (100%)	Vaccine, *n* = 8	Placebo, *n* = 7	Vaccine + IFNα-2a,*n* = 6	Placebo + IFNα-2a*, n* = 8
Median age (IQR)	46 (41–50.5)	49 (43–55.5)	47 (42–49)	43 (38.5–48.25)	44.5 (40–50.25)
Male	29 (100%)	8	7	6	8
Men who have sex with men (MSM)	27 (93%)	8	5	6	8
CD4 count (cells/mm^3^) at screening (median/IQR)	752 (705–1018)	751 (687–1206)	726 (642–1228)	933 (671–1203)	806 (708–864)
CD4 count (cells/mm^3^) after last vaccine − before ATI* (median/IQR)	739 (630–991)	790 (546–1542)	739 (614–1196)	770 (591–929)	721 (656–899)
CD4 count (cells/mm^3^) before restart ART^¥^ (median/IQR)	697 (488–768)	599 (488–744)	767 (489–816)	505 (459–644)	733 (561–828)

*ATI, Analytical Treatment Interruption; ^¥^ART, Antiretroviral Treatment.

Since all randomized participants interrupted ART in order to obtain the study products, all were included in the safety analysis. A total of 154 adverse events (AE) were reported, and we divided them into two stages, since screening and related to vaccine production and during vaccination until the end of study (**Table 2**). The most frequent AE related to DCV3 was inguinal lymphadenopathy and that regarding IFNα-2a was asthenia. No laboratory changes related to the interventions were considered to be grade 3 or 4. Overall, the procedures and the vaccines were safe and well tolerated. Of note, there was one participant who received placebo + IFNα-2a and had a very mild rash that we considered an allergic reaction to IFN, so we decided to stop this treatment after the first dose. Another participant who was allocated to study group 2 and received DCV3 + IFNα-2a presented with fever, asthenia, and myalgia that lasted 24 h after injecting each one of the first two doses of IFNα-2a. After the third dose, symptoms persisted and also referred dyspnea. A chest x-ray showed a left lung apical cavitation and sputum smear reported fast-acid bacilli. Quantiferon resulted negative and we started antimicrobial therapy for non-tuberculous mycobacteria. Lowenstein culture was positive for *Mycobacteria malmoense*. The participant has been followed up since by a multidisciplinary team. This patient was excluded from all other endpoint analysis since the study team considered that the referred mycobacterial infection would significantly affect the immune response and viral dynamics.

**Table 2:** Adverse events represented in two stages: Stage 1 before vaccination (w−52–w−1), and Stage 2 during vaccination and until the end of study (w0–w28) divided by severity and its relation to the interventions.

STAGE 1	RELATED				UNRELATED			
GRADE 1-2	27				41			
GRADE 3-4	0				3			
STAGE 2	DCV3	DCV3 + IFNα-2a	PLACEBO	PLACEBO + IFNα-2a	DCV3	DCV3 + IFNα-2a	PLACEBO	PLACEBO + IFNα-2a
GRADE 1-2	11	16	6	9	16	8	4	11
GRADE 3-4	0	1	0	0	0	0	1	0
STAGE 1	No AE related				Seminoma, Subarachnoid hemorrhage, Cataract surgery			
STAGE 2	*M. malmoense* pneumonia				Ischemic stroke			

At the end, a description of grade 3–4 adverse events accordingly is included.

IFN, Interferon.

### Effect of Vaccination on Viral Dynamics

**Figure 3:**
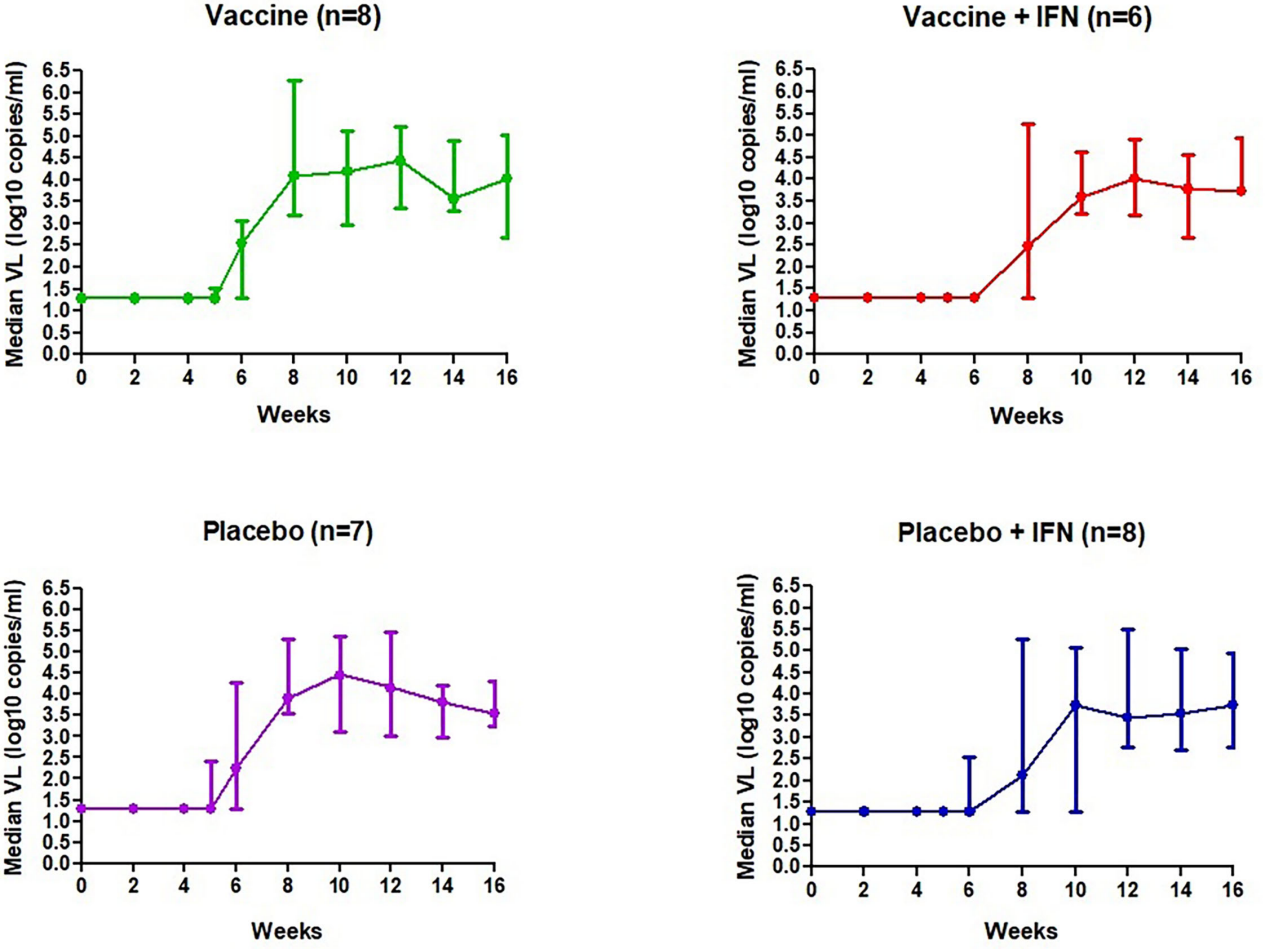
Median VL (log_10_ copies/ml) during vaccination and ATI per study group.

We analyzed the viral dynamics of 28 participants. All participants who received study products interrupted ART and rebounded before 12 weeks; median (range) time to rebound was 27 days (7-56), being significantly longer in the groups receiving IFNα-2a independently than those receiving DCV3 or placebo (36 vs. 19, *p* < 0.0001). Seven participants met protocol criteria to restart ART before the protocol-defined end of the ART interruption phase at study week 16. All but four participants resumed ART due to primary endpoint. Three individuals decided to continue without ART for one more week and one individual decided to continue without ART for four more weeks. Median VL during vaccination and ATI per study group are shown in **Figure 3**. At the end of the study and 3 months after restarting ART, all participants had a fully suppressed pVL and no evidence of emerge of drug resistance was detected.

**Figure 4:**
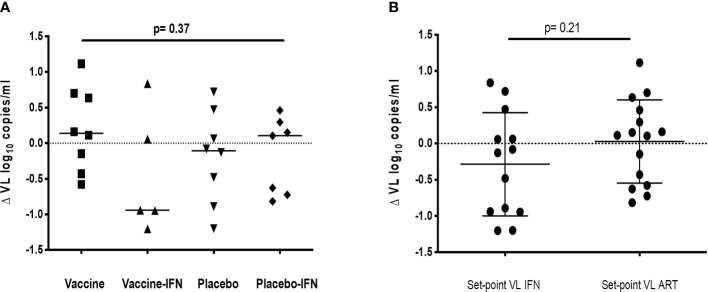
ΔVL set-point [log_10_ mean (SE) copies/ml] for different groups of study. **(A)** ΔVL set-point for patients receiving vaccine, vaccine + IFN, placebo and placebo + IFN; **(B)** ΔVL set-point for patient receiving IFN or not, independently of being vaccinated or not.

Set-point pVL [log_10_ mean (SE) copies/ml] in each group after intervention was: DCV3 4.33 (0.21), DCV3+IFNα-2a 3.85 (0.28), Placebo 4.15 (0.30), and Placebo+ IFNα-2a 3.87 (0.28). We compare differences by group between pre-ART set-point pVL and during ATI after vaccination {ΔpVL set-point [log10 mean (SE) copies/ml]: DCV3 0.20 (0.21), DCV3+IFNα-2a -044 (0.38), Placebo 0.17 (0.20) and Placebo+ IFNα-2a 0.19 (0.22)} and found a difference >0.5 log_10_ in 1, 3, 2, and 3 participants and a difference >1log_10_ in 0, 3, 1, and 0 participants from DCV3, DCV3+IFNα-2a, Placebo, and Placebo+ IFNα-2a, respectively (**Figure 4A**). When comparing between groups, we found a significant difference (>1log_10_ copies/ml) between DCV3 vs. DCV3+IFNα-2a and Placebo vs. DCV3+IFNα 2a (*p* = 0.03 and *p* = 0.04, respectively).

**Figure 5:**
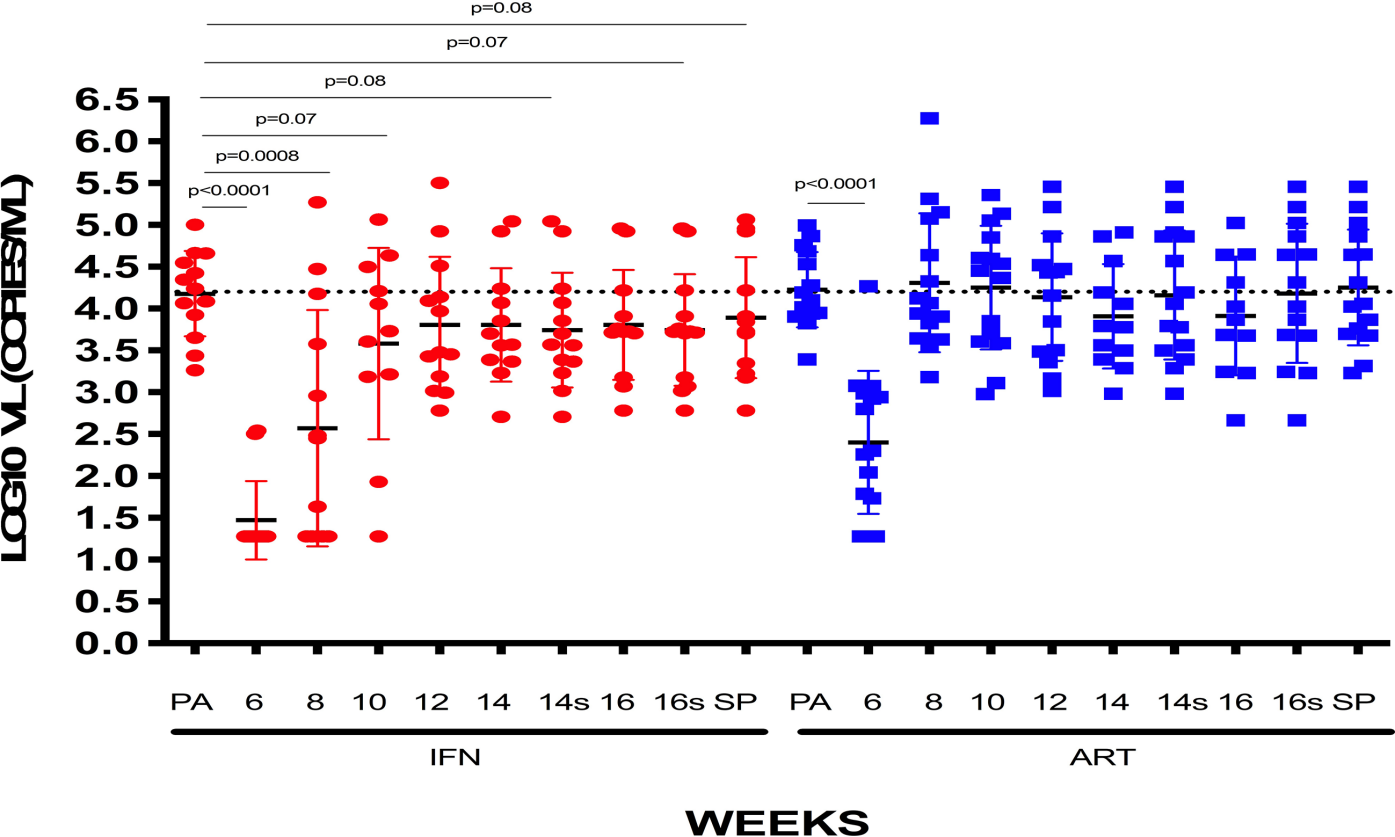
VL [log_10_ mean (SE) copies/ml] of patients receiving or not IFN: VLs at pre-ART, weeks 6, 8, 10, 12, 14, and 16 of follow-up and set-point are represented for patients who received IFN or not, independently of being previously vaccinated.

When analyzing the IFNα-2a effect on VL set-point, we did not find any difference regardless if combined with vaccine or placebo (**Figure 4B**). Viral dynamics comparing IFNα-2a-based strategies with no-IFNα-2a are shown in **Figure 5**.

### Effects on the HIV-1 Reservoir

All participants had detectable viral reservoirs throughout the study measured as total and integrated HIV-1 DNA in isolated CD4^+^ T cells. At screening (w−52), median (range) total HIV-1 DNA was 680.89 copies/10^6^ (23.54–6252.80), and at the end of the study (w28), 1029.44 copies/10^6^ (47.07–7472.71) without significant differences (*p* = 0.22); on the contrary, there was a significant increase on integrated HIV-DNA between screening and end of study [233.02 copies/10^6^ (8.50–1,312.27) vs. 434.18 (4.08–2,976.31) *p* = 0.009]. No significant differences were found in proviral DNA.

### Immunogenicity: Changes in HIV-1-Specific T-Cell Responses After Vaccination

**Figure 6:**
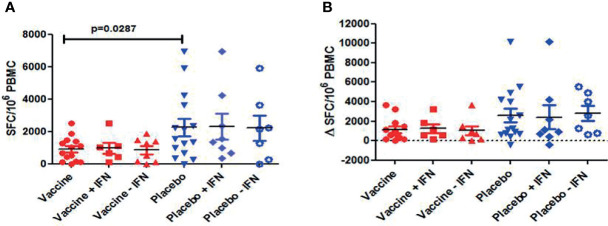
Total HIV-1 specific T cell responses measured by IFN-γ ELISPOT: **(A)** Individual responses and mean ± SEM of SFC/106 PBMC for the different arms of the study at w0 (baseline); **(B)** Changes of HIV-1-specific T cell responses at w16 for the different arms of the study [Δ SFC/106 PBMC (w16-w0)]. No significant differences were observed between vaccine and placebo groups (p=0.09). No effect on HIV-1-specific T cell responses was observed with the administration of IFN.

**Figure 7:**
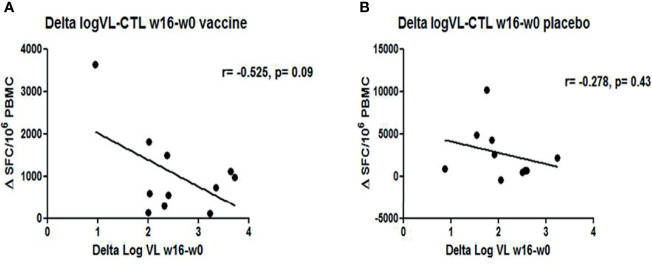
Correlations between changes in VL and HIV-1-specfic T-cell responses at w16: Graphs represent the trend of inverse correlation between ΔVL and ΔHIV-1-specific T cell responses (w16-w0) **(A)** in vaccine group (r=-0.525, p= 0.09) **(B)** whereas no correlation was observed in placebo group (r=-0.275, p= 0.09).

Immunogenicity was assessed in all subjects using IFN-γ ELISPOT at screening (w−52), w−12, w2, w4, w8, w16, and at end-of study (w28). At baseline, HIV-1-specific T-cell responses were significantly lower in DCV3 than in placebo groups [mean (SE) 900 (200) vs. 2,259 (535) SFC/10^6^ PBMC, *p* = 0.028]. The magnitude of responses showed variation over time but differences between vaccines and placebos were not statistically significant. Changes in the HIV-1-specific T-cell responses by groups and between different timings are exposed in **Table 3**. No significant differences in changes between w16 and baseline of HIV-1-specific T-cell responses were observed between vaccine and placebo groups (*p* = 0.009). Moreover, we did not observe any effect on specific T-cell responses with IFNα-2a administration (**Figure 6**). HIV-specific T-cell responses observed at week 16 tended to be inversely associated with the drop in pVL in vaccine group (*r* = −0.56, *p* = 0.08), while no significative correlation was observed in placebo group (*r* = −0.28, *p* = 0.43) (**Figure 7**).

**Table 3:** Changes in the HIV-1-specific T-cell responses by groups and between different timings.

Changes in HIV-specific responses [median (IQR)]						
	DCV3 (groups 1 and 3)			Placebo (groups 2 and 4)		*p*
Week 4	210 (12.50–1,308)			1,480 (310–2,363)		0.08
Week 8	280 (−71.50–939)			535 (−45–1,493)		0.64
Week 16^^^	660 (169.5–1740)			1,260 (675–4,220)		0.08
	DCV3	Placebo	*p*	DCV3+IFNα-2a	Placebo+IFNα-2a	*p*
Week 4	565 (49.75–1,541)	1,950 (1,120–2,363)	**0.04**	135 (−43.5–1,107)	352.5 (124.5–2,172)	0.35
Week 8	617.5 (2.50–1,348)	1,020 (−230–1,800)	0.53	110 (−91.5–587.5)	350 (−11.75–831)	0.62
Week 16^^^	735 (113–1,500)	2,568 (808–4,905)	0.07	585 (342.5–2,530)	1,033 (502.5–3,715)	0.62
	DCV3	DCV3+IFNα-2a	*p*	Placebo	Placebo+IFNα-2a	*p*
Week 4	565 (49.75–1,541)	135 (−43.50–1,107)	0.52	1,950 (1,120–2,363)	352.5 (124.5–2,172)	0.23
Week 8	617.5 (2.50–1,348)	110 (−91.5–587.5)	0.22	1,020 (−230–1,800)	350 (−11.75–831)	0.33
Week 16^^^	735 (113–1,500)	585 (342.5–2,530)	0.75	2568 (808–4,905)	1,033 (502.5–3,715)	0.39

Bold means statistically significant.

## Discussion

The combination of an intranodally administered MD-DC therapeutic vaccine and subcutaneous IFNα-2a was overall safe and well tolerated in PLWH on ART. However, we did not find any significant impact on viral dynamics from this intervention.

Almost all of the adverse events reported during the trial were mild and tolerable, even during the ultrasound-guided intranodal administration. One participant was excluded from the immunogenicity and viral dynamics analysis because during treatment interruption, he suffered a *M. malmoense* pneumonia. To date, we cannot discard the effect of our interventions in the reactivation of this infection. The detailed case report has been submitted elsewhere.

Our study showed a very modest decrease in set-point VL in vaccine recipients and it was correlated with an increase of HIV-1-specific T-cell responses. Baseline HIV-1-specific T-cell responses were significantly higher in the placebo group, and although we could hypothesize that this means an overall better functional response, this is not appreciated during the study. Even if the increase in specific T-cell responses was moderated, as measured by Elispot at week 16 after ART interruption, the pVL in immunized patients tend to be inversely correlated with HIV-1-specific T-cell responses, whereas no correlation was observed in the placebo group. All these results suggest that pulsed-DC could probably be able to appropriately stimulate T cells in order to try to obtain an efficient immune response. A systematic review of DC-based strategies concluded that although the response to HIV *in vitro* is very promising, the clinical outcomes have been modest, and opportunities to improve the efficacy of this strategy remain (11). Type I IFNs modulate cellular antiviral immune responses *in vivo*, either directly by activation of antiviral host restriction factors or indirectly *via* stimulation of innate NK cell-mediated responses. However, and even if it has been observed that IFNα can boost CD8^+^ T-cell responses (17), our results did not show any influence of IFNα-2a on the HIV-specific T-cell responses.

In PLWH, the potential for IFN-α2a immunotherapy to reduce the size of the HIV reservoir (measured as the integrated HIV DNA) in chronic ART-suppressed HIV or hepatitis C virus (HCV) co-infected individuals has been previously described (16, 21, 22). We conducted a sub-study to assess if transcriptomics and metagenomics could be used as surrogate markers of the vaccines’ response, and one of our main findings was that both omics data were associated to the VL response during ATI. In this study, participants with a decline of >0.5log_10_ copies/ml in viral set-point during ATI compared to pre-ART were considered as “responders” and had enriched levels of the IFI27 and IFI6 genes involved in the defense response to the virus and type 1 IFN signaling (23). In our study, we found no significant effect of IFNα-2a in viral dynamics, possibly related to the short-course treatment of just three doses as compared to longer treatment in previous studies.

Our study has limitations. Even if most of the baseline characteristics of the participants were similar to other clinical trials, the design of our study (including route of administration, type of immunogen, number of doses, and schedule) varies for most of them, so it is challenging to analyze them in comparison. Another challenge in interpreting our results is having a small sample size; although there is a possibility, it is not entirely clear that having more participants could mean a better outcome. Our previous experience with a DC-based vaccine was very positive; there were significant changes in VL set-point that were clearly associated with an increase in HIV-1 T-cell responses (13); in our current study, we did not find this effect even though we used a more favorable route of administration and in combination with an immunomodulatory agent, which makes us think that it could be related to the vaccine itself since it was produced differently. It is also worth considering whether or not autologous virus is a good antigen. Although in our previously published clinical trials of DC-based vaccines as well as in our current study, our data strongly suggest that a combination of autologous antigens administered to PLWH on ART could have a higher virological effect, it should be considered that the source of the autologous virus could also have an impact on the virological outcome. In a previous trial performed by our group (13), the autologous inactivated HIV-1 antigen was prepared fulfilling clinical-grade good manufacturing practice (cGMP) requirements, and the primary virus isolate was obtained and propagated by means of co-culture of CD4-enriched PBMCs from both the person with HIV-1 and heterologous CD4^+^ T cells from healthy donors (24). Those viruses were considered partially autologous because they had the envelope that could include heterologous proteins and aggregates or vesicles from the healthy donor. By contrast, in our current trial, the autologous HIV-1 antigen was produced in co-cultures with autologous macrophages, which could contain other compounds (microvesicles, protein aggregates released by macrophages) that could confer some degree of immune tolerance and then be less immunogenic. This fact could explain the lowest impact on the HIV-specific T-cell responses with a consequent lower effect on the control of VL. More studies to compare both techniques could be enlightening.

In the current study, we have selected the intranodal route for vaccine administration since we wanted to determine the direct effect of this approach as a major boost of vaccination regimen and to ensure that all the HIV-pulsed DC will exert effects on this highly immune-activating tissue. There is no previous experience administrating MC-DC intranodally in the HIV field, but it has been used in cancer with encouraging results and the authors believe that it is a more reliable and faster method to obtain a positive outcome since, by other routes, only a small percentage of the DC administered reach the lymph nodes (25–27). Nevertheless, the limited effect on VL dynamics, viral reservoir, and HIV-specific T-cell responses did not allow us to make strong conclusions about this route in HIV DC vaccines.

In conclusion, the combination of MD-DC therapeutic vaccine administered intranodally and IFNα 2a was safe and had a very mild impact in viral dynamics and T-cell-specific responses. Considering all previous findings regarding DC vaccines, there is still room for improvement, and an overhaul of vaccine development, routes, doses, and combining therapies is needed.

## Data Availability Statement

The data that support the findings of this study are available from the corresponding author, upon reasonable request.

## Ethics Statement

The studies involving human participants were reviewed and approved by Comité de Ética de la Investigación en Medicamentos (CEim) del Hospital Clínic Barcelona. The patients/participants provided their written informed consent to participate in this study.

## DCV3-RISVAC04 Study Group

Lorna Leal, Irene Fernández, Elvira Couto, Yolanda Romero, Laia Miralles, Carmen Hurtado, Núria Climent, Tania González, Maria José Maleno, Judit Pich, Laura Burunat, Joan Albert Arnaiz, Blanca Paño, Carlos Nicolau, Rafael Salvador, Elisabet Farré, Sonsoles Sánchez-Palomino, José Maria Gatell, Montserrat Plana, Felipe García, Berta Torres, Constanza Lucero, Montserrat Laguno, María Martínez-Rebollar, Ana González-Cordón, John Rojas, Alexy Inciarte, Lorena de la Mora, Josep Mallolas, Esteban Martínez, José Luis Blanco, Unai Perpiñá, Josep Canals, Raquel Martín, Florencia Echeverry, Cristina Xufré, Cristina Rovira, Marta Sala, and Amparo Tricas.

## Author Contributions

LL, JG, MP, and FG conceived and designed the study. SS-P, NC, and JP contributed to the study design. EC, YR, and IF contributed to data management. LL, EC, LM, MM, TG, BP, and CN performed the experiments. LL, SS-P, MP, and FG undertook the statistical analysis. LL, MP, and FG drafted the manuscript. SS-P, NC, and JG participated in study analyses and revised the manuscript critically for important intellectual content. All authors contributed to the article and approved the submitted version.

## Funding

This study was partially supported by grants from the Spanish Ministry of Economy (MINECO) (grants: SAF2015-66193-R, SAF-2017-88089-R, and RTI2018-096309-B-I00); the Fondo Europeo para el Desarrollo Regional (FEDER); the SPANISH AIDS Research Network RD16/0025/0002 and RD16/0025/0014-ISCIII-FEDER (RIS); the Fondo de Investigación Sanitaria (FIS) AC16/00051, PI12/01247, and PI18/00699; the Instituto de Salud Carlos III (grants: COV20/00214 and ICI20/00067); HIVACAT program; and the CERCA Programme/Generalitat de Catalunya SGR 615 and SGR 653. This manuscript was funded by the European Commission [grant numbers: FP7-HEALTH-2013-INNOVATION-1 602570-2 and H2020-SC1-2016-2017 (H2020-SC1-2016-RTD) Proposal: 731626-HIVACAR].

## Conflict of Interest

JG has received honoraria for speaking and advisory boards, and his institution has received research grants from ViiV, MSD, Janssen, and Gilead. As of May 1, 2018, JG is a full-time employee of ViiV Healthcare.

The authors declare that the research was conducted in the absence of any commercial or financial relationships that could be construed as a potential conflict of interest.

## Publisher’s Note

All claims expressed in this article are solely those of the authors and do not necessarily represent those of their affiliated organizations, or those of the publisher, the editors and the reviewers. Any product that may be evaluated in this article, or claim that may be made by its manufacturer, is not guaranteed or endorsed by the publisher.

## References

[1] Palella FJ Kathleen M Delaney M Holmberg S. Declining Morbidity and Mortality Among Patients With Advanced Human Immunodeficiency Virus Infection. N Engl J Med (1998) 338:853–60. doi: 10.1056/NEJM199803263381301 9516219

[2] Beyrer C Pozniak A. HIV Drug Resistance — An Emerging Threat to Epidemic Control. N Engl J Med (2017) 377(17):1605–7. doi: 10.1056/NEJMp1710608 29069566

[3] Willig AL Overton ET. Metabolic Complications and Glucose Metabolism in HIV Infection: A Review of the Evidence. Curr HIV/AIDS Rep (2016) 13(5):289–96. doi: 10.1007/s11904-016-0330-z PMC5425100 27541600

[4] García F. ‘Functional Cure’ of HIV Infection: The Role of Immunotherapy. Immunotherapy (2012) 4:245–8. doi: 10.2217/imt.12.2 22401627

[5] Gamberg J Lambert L Bowmer M Howley C GM. Factors Related to Loss of HIV−specific Cytotoxic T Lymphocyte Activity. AIDS (2004) 18:597–604. doi: 10.1097/00002030-200403050-00003 15090764

[6] Siliciano JD Kajdas J Finzi D Quinn TC Chadwick K Margolick JB. Long-Term Follow-Up Studies Confirm the Stability of the Latent Reservoir for HIV-1 in Resting CD4+ T Cells. Nat Med (2003) 9(6):727–8. doi: 10.1038/nm880 12754504

[7] Barton K Hiener B Winckelmann A Rasmussen TA Shao W Byth K. Broad Activation of Latent HIV-1 *In Vivo* . Nat Commun (2016) 7:12731. doi: 10.1038/ncomms12731 27605062 PMC5025526

[8] Deeks SG Lewin SR Ross AL Ananworanich J Benkirane M Cannon P. International AIDS Society Global Scientific Strategy: Towards an HIV Cure 2016. Nat Med (2016) 22(8):839–50. doi: 10.1038/nm.4108 PMC5322797 27400264

[9] Leal L Lucero C Gatell JM Gallart T Plana M García F. New Challenges in Therapeutic Vaccines Against HIV Infection. Expert Rev Vaccines (2017) 16(6):587–600. doi: 10.1080/14760584.2017.1322513 28431490

[10] Cintolo JA Datta J Mathew SJ Czerniecki BJ. Dendritic Cell-Based Vaccines: Barriers and Opportunities. Futur Oncol (2012) 8(10):1273–99. doi: 10.2217/fon.12.125 PMC4260651 23130928

[11] Campos Coelho AV de Moura RR Kamada AJ da Silva RC Guimarães RL Brandão LAC. Dendritic Cell-Based Immunotherapies to Fight HIV: How Far From a Success Story? A Systematic Review and Meta-Analysis. Int J Mol Sci (2016) 17(12):1985. doi: 10.3390/ijms17121985 PMC5187785 27898045

[12] García F Routy JP. Challenges in Dendritic Cells-Based Therapeutic Vaccination in HIV-1 Infection. Workshop in Dendritic Cell-Based Vaccine Clinical Trials in HIV-1. Vaccine (2011) 29(38):6454–63. doi: 10.1016/j.vaccine.2011.07.043 21791232

[13] García F Climent N Guardo AC Gil C León A Autran B. A Dendritic Cell-Based Vaccine Elicits T Cell Responses Associated With Control of HIV-1 Replication. Sci Transl Med (2013) 5(166):166ra2. doi: 10.1126/scitranslmed.3004682 23283367

[14] Pantaleo G Lévy Y. Vaccine and Immunotherapeutic Interventions. Curr Opin HIV AIDS (2013) 8(3):236–42. doi: 10.1097/COH.0b013e32835fd5cd 23478912

[15] Pantaleo G Levy Y. Therapeutic Vaccines and Immunological Intervention in HIV Infection: A Paradigm Change. Curr Opin HIV AIDS (2016) 11(6):576–84. doi: 10.1097/COH.0000000000000324 27607591

[16] Azzoni L Foulkes AS Papasavvas E Mexas AM Lynn KM Mounzer K. Pegylated Interferon Alfa-2a Monotherapy Results in Suppression of HIV Type 1 Replication and Decreased Cell-Associated HIV DNA Integration. J Infect Dis (2013) 207(2):213–22. doi: 10.1093/infdis/jis663 PMC3532820 23105144

[17] Le Bon A Etchart N Rossmann C Ashton M Hou S Gewert D. Cross-Priming of CD8+ T Cells Stimulated by Virus-Induced Type I Interferon. Nat Immunol (2003) 4(10):1009–15. doi: 10.1038/ni978 14502286

[18] Alfaro C Perez-Gracia JL Suarez N Rodriguez J Fernandez de Sanmamed M Sangro B. Pilot Clinical Trial of Type 1 Dendritic Cells Loaded With Autologous Tumor Lysates Combined With GM-CSF, Pegylated IFN, and Cyclophosphamide for Metastatic Cancer Patients. J Immunol (2011) 187(11):6130–42. doi: 10.4049/jimmunol.1102209 22048768

[19] U.S. Department of Health and Human Services National Institutes of Health National Institute of Allergy and Infectious Diseases D of A. USA: Division of AIDS. National Institute of Allergy and Infectious Diseases. National Institutes of Health US Department of Health and Human Services (2014). Available at: https://rsc.niaid.nih.gov/sites/default/files/daids-ae-grading-table-v2-nov2014.pdf.

[20] Vandergeeten C Fromentin R Merlini E Lawani MB DaFonseca S Bakeman W. Cross-Clade Ultrasensitive PCR-Based Assays To Measure HIV Persistence in Large-Cohort Studies. J Virol (2014) 88(21):12385–96. doi: 10.1128/jvi.00609-14 PMC4248919 25122785

[21] Jiao YM Weng WJ Gao QS Zhu WJ Cai WP Li LH. Hepatitis C Therapy With Interferon-α and Ribavirin Reduces the CD4 Cell Count and the Total, 2LTR Circular and Integrated HIV-1 DNA in HIV/HCV Co-Infected Patients. Antiviral Res (2015) 118:118–22. doi: 10.1016/j.antiviral.2015.03.011 25823618

[22] Sun H Buzon MJ Shaw A Berg RK Yu XG Ferrando-Martinez S. Hepatitis C Therapy With Interferon-α and Ribavirin Reduces CD4 T-Cell-Associated HIV-1 DNA in HIV-1/Hepatitis C Virus-Coinfected Patients. J Infect Dis (2014) 209(9):1315–20. doi: 10.1093/infdis/jit628 PMC3982848 24277743

[23] Pastor-Ibáñez R Díez-Fuertes F Sánchez-Palomino S Alcamí J Plana M Torrents D. Impact of Transcriptome and Gut Microbiome on the Response of Hiv-1 Infected Individuals to a Dendritic Cell-Based Hiv Therapeutic Vaccine. Vaccines (2021) 9(7):694. doi: 10.3390/vaccines9070694 34202658 PMC8310021

[24] Gil C Climent N García F Hurtado C Nieto-Márquez S León A. Ex Vivo Production of Autologous Whole Inactivated HIV-1 for Clinical Use in Therapeutic Vaccines. Vaccine (2011) 29:5711–24. doi: 10.1016/j.vaccine.2011.05.096 21679735

[25] Morisaki T Morisaki T Kubo M Onishi H Hirano T Morisaki S. Efficacy of Intranodal Neoantigen Peptide-Pulsed Dendritic Cell Vaccine Monotherapy in Patients With Advanced Solid Tumors: A Retrospective Analysis. Anticancer Res (2021) 41(8):4101–15. doi: 10.21873/anticanres.15213 34281881

[26] Bedrosian I Mick R Xu S Nisenbaum H Faries M Zhang P. Intranodal Administration of Peptide-Pulsed Mature Dendritic Cell Vaccines Results in Superior CD8+ T-Cell Function in Melanoma Patients. J Clin Oncol (2003) 21(20):3826–35. doi: 10.1200/JCO.2003.04.042 14551301

[27] Aarntzen EHJG Schreibelt G Bol K Lesterhuis WJ Croockewit AJ De Wilt JHW. Vaccination With mRNA-Electroporated Dendritic Cells Induces Robust Tumor Antigen-Specific CD4+ and CD8+ T Cells Responses in Stage III and IV Melanoma Patients. Clin Cancer Res (2012) 18(19):5460–70. doi: 10.1158/1078-0432.CCR-11-3368 22896657

